# Knockdown of GTPBP4 inhibits cell growth and survival in human hepatocellular carcinoma and its prognostic significance

**DOI:** 10.18632/oncotarget.21500

**Published:** 2017-10-05

**Authors:** Wen-Bin Liu, Wei-Dong Jia, Jin-Liang Ma, Ge-Liang Xu, Hang-Cheng Zhou, Yan Peng, Wei Wang

**Affiliations:** ^1^ Department of Hepatic Surgery, Anhui Provincial Hospital, Anhui Medical University, Hefei 230001, P.R. China; ^2^ Anhui Province Key Laboratory of Hepatopancreatobiliary Surgery, Hefei 230001, P.R. China; ^3^ Department of Pathology, Anhui Provincial Hospital, Anhui Medical University, Hefei 230001, P.R. China; ^4^ Department of Medical Oncology, Anhui Provincial Hospital, Anhui Medical University, Hefei 230001, P.R. China

**Keywords:** GTPBP4, hepatocellular carcinoma, oncogene, prognosis, therapeutic target

## Abstract

GTP-binding protein 4 (GTPBP4), as a novel member of GTPases involved in the synthesis of 60S subunit and maturation, is closely related to cell proliferation and growth. Till now, a small number of existing studies have found a contradictory dual role of GTPBP4 in cancer. Whether the expression level of GTPBP4 in hepatocellular carcinoma (HCC) is associated with the patients’ prognosis or its function and underlying molecular mechanisms still remains unclear. In the present study, the above issues were explored for the first time. Our results showed that GTPBP4 was overexpressed in HCC and knockdown of GTPBP4 delayed cell proliferation, impaired colony formation ability, induced cell cycle arrest in G2/M period and promoted apoptosis in HCC cell lines. Besides, *in vivo* xenograft nude mice model revealed that GTPBP4 knockdown could significantly suppress HCC tumorigenesis. Gene microarray and further pathway enrichment analyses indicated that ERBB signaling pathway was the most significantly changed one. More importantly, high GTPBP4 expression level significantly correlated to the poor prognosis of HCC patients. Taken together, all these findings suggest that GTPBP4 serves as an oncogene and plays a pivotal role in HCC development, which will be a potential therapeutic target or a biomarker for HCC.

## INTRODUCTION

Liver cancer is one of the most common cancer type and the fifth leading cause of cancer-related death among men *in* the *United States* [1]. And hepatocellular carcinoma (HCC) is the major type and accounts for nearly 90% of primary liver cancer [2]. Most patients are diagnosed with HCC at advanced stages, resulting in HCC high mortality [3]. Though in recent years, the molecular characteristics of HCC have been explored broadly by the high-throughput sequencing methods [4, 5], biomarkers, which are crucial to HCC early diagnosis and effective targeted therapy, have remained rather unsatisfactory. Therefore, to explore the molecular pathways and potential therapeutic target underlying HCC tumorigenesis and progression is still urgently needed.

GTP-binding protein 4 (GTPBP4), also known as NGB, CRFG or NOG1, is a novel member of GTPases which belongs to the guanine nucleotide-binding proteins family [6-8]. GTPBP4 mainly locates in the nucleolus [9], which is involved in the synthesis of 60S subunit [10] and maturation [11], closely related to cell proliferation and growth. At present, there are few studies on this gene in tumor, but only a few literatures have indicated that it is related to glioma cell proliferation [8], breast cancer patients’ prognosis [12] and colorectal cancer metastasis [13]. However, the different expression levels of GTPBP4 (downregulated or upregulated) were found in those malignant tumors, suggesting that GTPBP4 may act as an oncogene or a suppressor gene mainly dependent upon the specific cancer type. Till now, the expression level and function of GTPBP4 in HCC has not been explored. Furthermore, whether GTPBP4 can be used as a potential therapeutic target for HCC also needs to be investigated.

Just recently, bioinformatics methods were used to reveal that GTPBP4 mRNA level was higher in HCC tissues than that in normal control group by mining the Oncomine and other databases. To testify and explain this phenomenon, GTPBP4 expression levels in HCC cell lines and tissues were examined in the present study. Then, the prognostic significance of GTPBP4 expression in HCC patients was explored. Further, lentivirus-mediated short-hairpin RNA (shRNA) targeting of GTPBP4 was used to investigate the role of its silencing on the proliferation, colony formation, cell cycle progression and apoptosis of HCC cells. An *in vivo* xenograft nude mice model was established to detect the effect of GTPBP4 knockdown on HCC tumorigenesis. Moreover, the underlying molecular mechanisms of GTPBP4 in HCC were analyzed by global gene profiling.

## RESULTS

### GTPBP4 was highly expressed in human HCC cell lines and tissues

**Figure 1:**
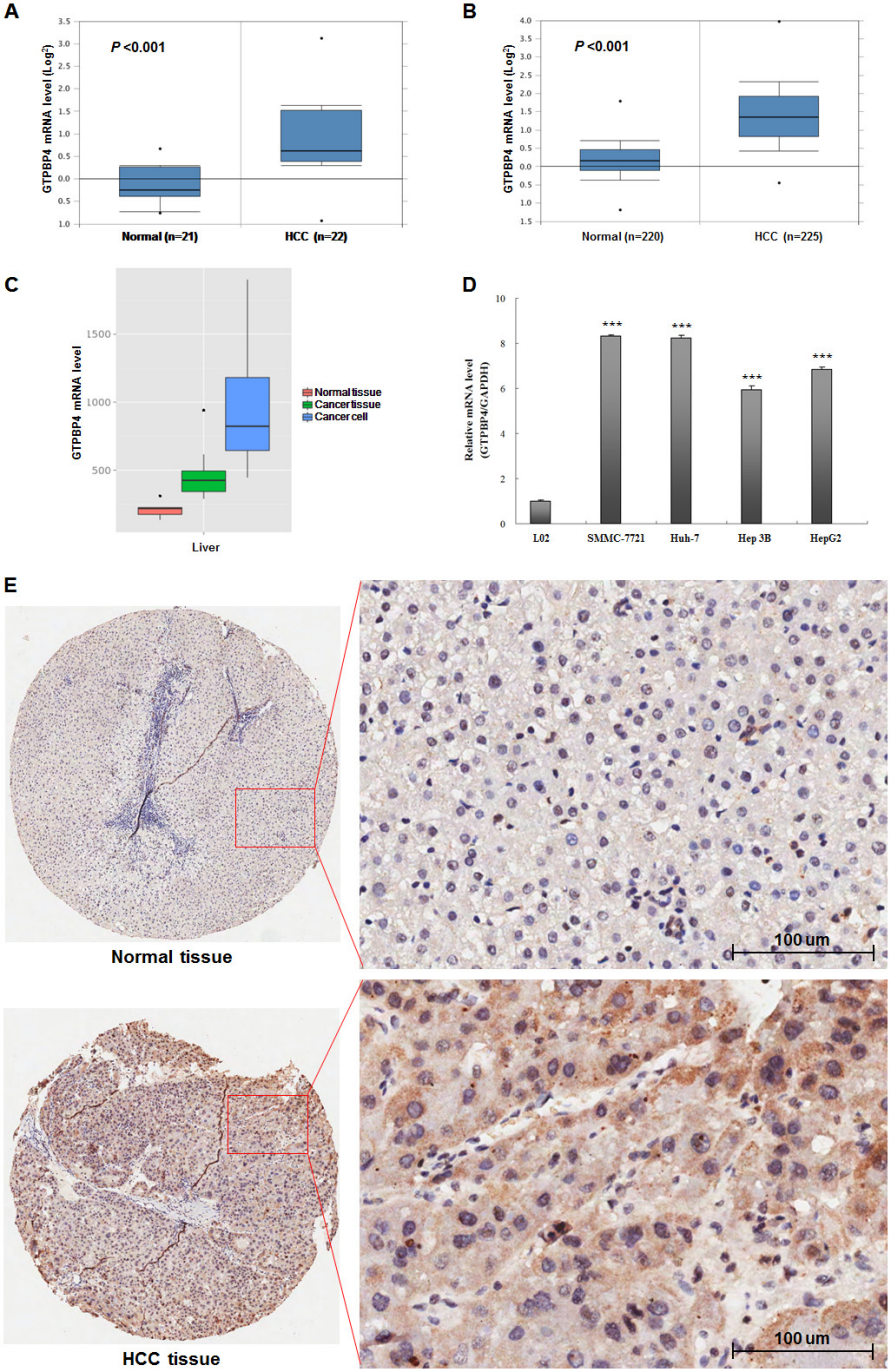
High expression levels of GTPBP4 mRNA and protein in HCC Oncomine database mining analysis of GTPBP4 mRNA levels in **(A)** Roessler Liver (GEO: GSE 14520/GPL571) and **(B)** Roessler Liver2 (GEO: GSE 14520/GPL3921) grouped by HCC and normal liver; **(C)** MERAV database mining analysis of GTPBP4 mRNA levels in normal liver tissues, primary liver tumor tissues, and liver cancer cells, respectively; **(D)** relative mRNA expression levels of GTPBP4 in four HCC cell lines (SMMC-7721, Huh 7, Hep 3B and HepG2) and normal liver cell line (L02) detected by qPCR; **(E)** protein levels of GTPBP4 in 90 cases of HCC tissues and paired adjacent normal liver tissues detected by immunochemistry. ^***^P < 0.001.

Initially, Oncomine database (https://www.oncomine.org/resource/login.html) was used to predict the GTPBP4 mRNA expression level in HCC and normal tissues. Compared to the normal group, the expression level of GTPBP4 mRNA was dramatically higher in HCC tissues (*P* <0.001, Figure 1A and 1B). Besides, the similar result was found through MERAV database [14] (http://merav.wi.mit.edu/SearchByGenes.html) as shown in Figure 1C. Next, in order to verify the above predictive findings, four HCC cell lines (SMMC-7721, HepG2, Hep3B and Huh-7) and 90 cases of HCC tissues was chosen to examine the expression levels of GTPBP4 mRNA and protein, respectively. Compared to the corresponding normal group (L02 hepatic cell line and the matched adjacent normal HCC tissues), either mRNA or protein expression level of GTPBP4 in HCC cell lines (Figure 1D) or HCC tissues (Figure 1E) was remarkably higher (all *P* values <0.05), respectively. The representative HE images of HCC and normal tissues were shown as the Supplementary Figure 1.

### High expression of GTPBP4 was closely associated with poor prognosis of HCC patients

**Figure 2:**
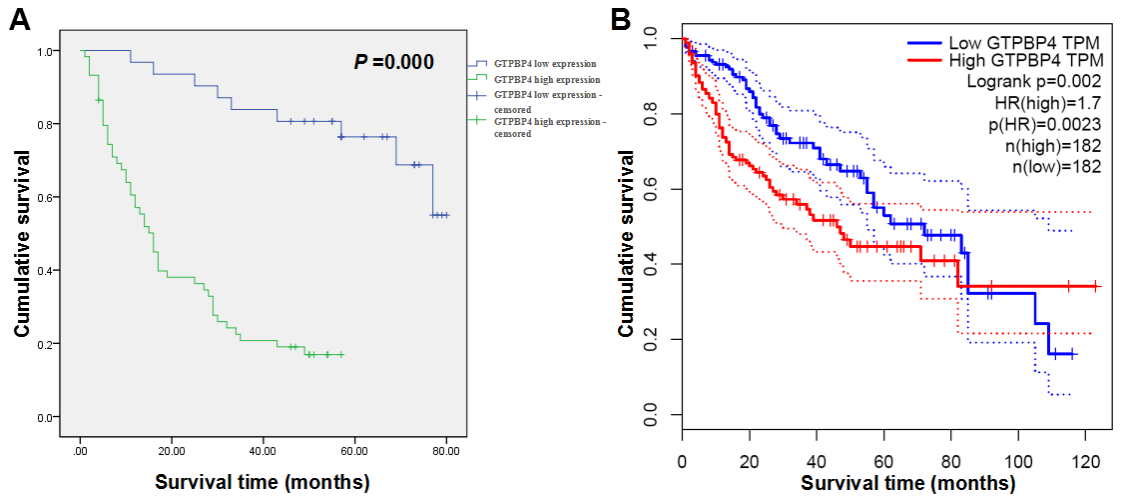
High expression levels of GTPBP4 protein and mRNA remarkably predicted the poor overall survival time of HCC patients in Kaplan-Meier survival analysis **(A)** Kaplan-Meier survival curve on the relationship between GTPBP4 protein expression and OS of 90 cases of HCC patients collected in the study; **(B)** Kaplan-Meier survival curve on the relationship between GTPBP4 mRNA expression and OS of HCC patients predicted by OncoLnc database (data from TCGA database).

To explore whether the expression of GTPBP4 was associated with the prognosis of HCC patients, the immunochemical results of HCC tissues were statistically analyzed further. The results showed that GTPBP4 staining was mainly located in the cytoplasm. GTPBP4 protein expression in HCC tissues was significantly higher than that in the paracarcinomatous tissues (Figure 1E). We evaluated the association between GTPBP4 protein expression and clinicopathological characteristics of HCC patients, including age, gender, Edmondson classification, tumor size, vascular invasion, cirrhosis and TNM stage (Table 1). The expression level of GTPBP4 protein in HCC was significantly associated with the TNM stage (*P* = 0.022). Next, the Kaplan-Meier method and log-rank test were performed to further analyze the overall survival (OS) rate of HCC patients with high or low GTPBP4 protein expression (Table 2). From the Kaplan-Meier survival curve, we observed that patients with high levels of GTPBP4 protein expression had significantly shorter survival time than those with low levels of GTPBP4 protein expression (*P* = 0.000, Figure 2A). Besides, tumor size, vascular invasion and TNM stage were also significantly associated with the OS of HCC patients (Table 2). Consistently, OncoLnc database [15] (http://www.oncolnc.org/, data from TCGA) showed that HCC patients with high expression level of GTPBP4 mRNA had worse prognosis than those with low GTPBP4 mRNA level (*P* = 0.002, Figure 2B). Furthermore, multivariate Cox regression analysis showed that high GTPBP4 protein expression in HCC was an independent prognostic factor for OS in HCC patients after curative resection (*P* = 0.000, Table 3). Thus, it was concluded that overexpression of GTPBP4 might be a novel biomarker for HCC prognosis and have a critical role in HCC progression and development.

**Table 1:** Correlation between GTPBP4 expression and clinicopathological characteristics in patients with hepatocellular carcinoma

Variables	Cases (N)	GTPBP4 expression		*P* value
		Low	High	
**Age at surgery (years)**				
≤ 60	66	21	45	0.385
> 60	24	10	14	
**Gender**				
Male	81	26	55	0.266
Female	9	5	4	
**Edmondson classification**				
I-II	57	20	37	0.866
III-IV	33	11	22	
**Tumor size (cm)**				
≤ 5	38	17	21	0.079
> 5	52	14	38	
**Vascular invasion**				
Yes	7	1	6	0.415
No	83	30	53	
**Cirrhosis**				
Yes	33	15	18	0.094
No	57	16	41	
**TNM stage**				
I-II	46	21	25	0.022
III-IV	44	10	34	

*P* values were calculated by χ2 test or Fisher’s exact test.

**Table 2:** Univariate analysis of the effects of GTPBP4 expression and clinicopathological characteristics on overall survival in patients with hepatocellular carcinoma

Variables	Mean survival time (months)	95% CI	*P* value
**GTPBP4 expression**			
High	22.298	17.417-27.178	0.000
Low	67.384	59.733-75.034	
**Age at surgery (years)**			
≤ 60	37.431	29.688-45.173	0. 261
> 60	47.917	36.072-59.762	
**Gender**			
Male	39.778	32.863-46.693	0.652
Female	41.556	22.570-60.541	
**Edmondson classification**			
I-II	41.856	33.717-49.996	0.493
III-IV	36.774	26.070-47.477	
**Tumor size (cm)**			
≤ 5	51.725	41.821-61.629	0.005
> 5	31.687	23.789-39.586	
**Vascular invasion**			
Yes	16.286	3.395-29.176	0.017
No	42.079	35.254-48.904	
**Cirrhosis**			
Yes	41.028	29.907-52.148	0.965
No	39.801	31.625-47.977	
**TNM stage**			
I-II	50.092	41.272-58.912	0.001
III-IV	29.639	21.018-38.259	

**Table 3:** Multivariate analysis of the effects of GTPBP4 expression and clinicopathological characteristics on overall survival in patients with hepatocellular carcinoma

Covariates	HR	95% CI	*P* value
GTPBP4 expression (low vs. high)	7.141	3.086-16.524	0.000
Tumor size (≤ 5 vs. >5 cm)	1.423	0.745-2.717	0.285
Cirrhosis (No vs.Yes)	2.302	0.945-5.607	0.066
TNM stage (stage I-II vs. III-IV)	1.441	0.783-2.653	0.240

### Efficient silencing of GTPBP4 expression in HCC cell lines

**Figure 3:**
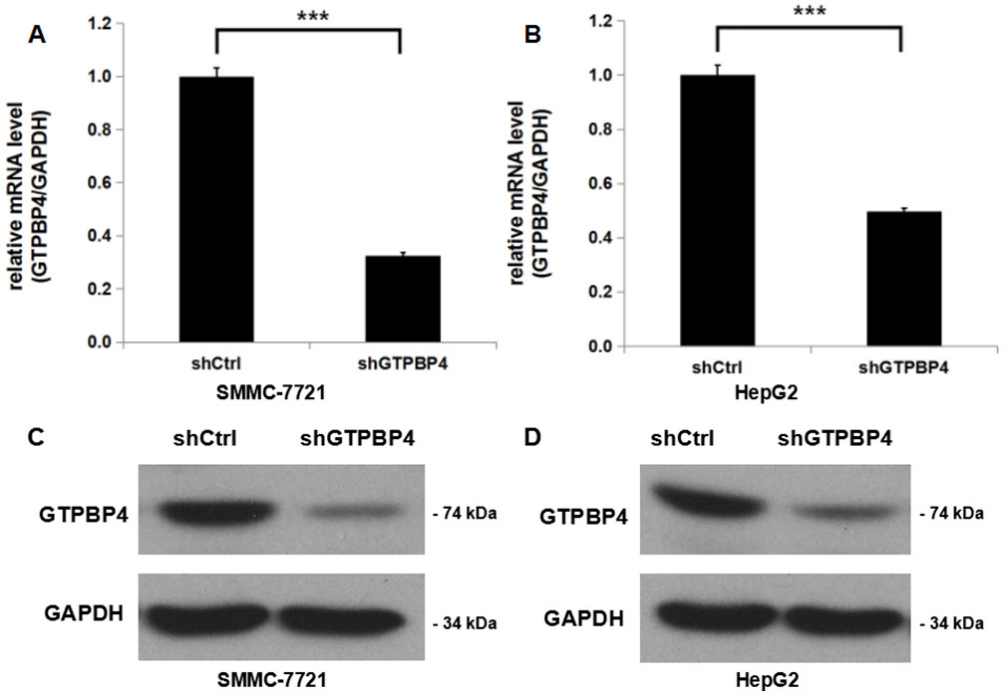
Efficient shRNA-mediated knockdown of GTPBP4 in HCC cell lines Cells of HCC cell lines were infected with lentiviruses expressing shRNA targeting GTPBP4. **(A)** GTPBP4 mRNA level in SMMC-7721 cells was knocked down efficiently by shRNA examined by qPCR; **(B)** GTPBP4 mRNA level in HepG2 cells was knocked down efficiently by shRNA examined by qPCR; The relative GTPBP4 protein levels in **(C)** SMMC-7721 and **(D)** HepG2 cells transfected with a plasmid expressing either Ctrl-shRNA or GTPBP4-shRNA were verified by western blot. ^***^P < 0.001.

To investigate the molecular functions of GTPBP4 in tumorigenesis of HCC, a lentivirus-mediated shRNA strategy was chosen to inhibit GTPBP4 expression in two human HCC cell lines (SMMC-7721 and HepG2). The gene silencing efficiency mediated by lentivirus was further determined using qPCR after culturing for 5 days, and it was shown that in both cell lines, GTPBP4 expression at mRNA level was inhibited significantly, with 67.5% knockdown efficiency observed in SMMC-7721 cells (Figure 3A) and 50.3% knockdown efficiency in HepG2 cells (Figure 3B). We also examined the GTPBP4 protein levels in SMMC-7721 and HepG2 cells after GTPBP4 knockdown by western blot, which were both remarkably inhibited (Figure 3C and 3D).

### Knockdown of GTPBP4 suppressed growth and colony formation, increased G2/M phase cell cycle arrest, and promoted apoptosis in HCC cell lines

**Figure 4:**
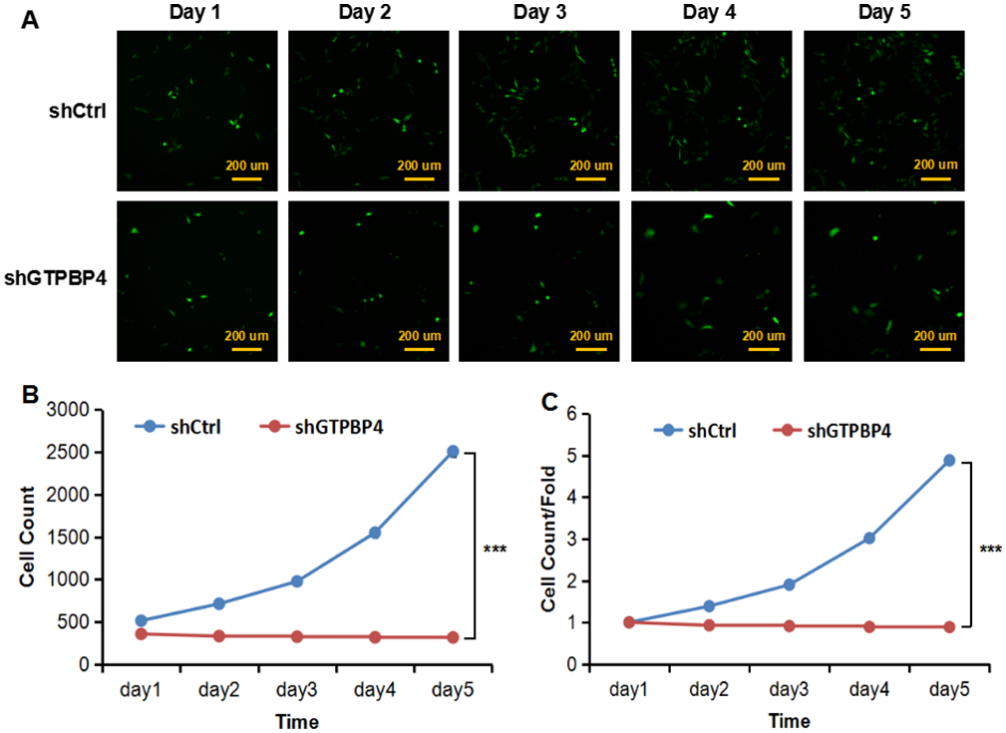
Knockdown of GTPBP4 suppressed cell proliferation in SMMC-7721 **(A)** Cell growth was detected by multiparametric high-content screening (HCS) every day for five days after lentivirus infection (bar =200 um); **(B)** cell numbers of SMMC-7721 were quantified by cellomics detection every day and the cell proliferation rate was analyzed; **(C)** fold changes of cell proliferation were also calculated between two groups. shCtrl: cells infected with non-targeting shRNA lentivirus; shGTPBP4: cells infected with GTPBP4-targeting shRNA lentivirus. Data were presented as means ± SD, ^***^P <0.001.

**Figure 5:**
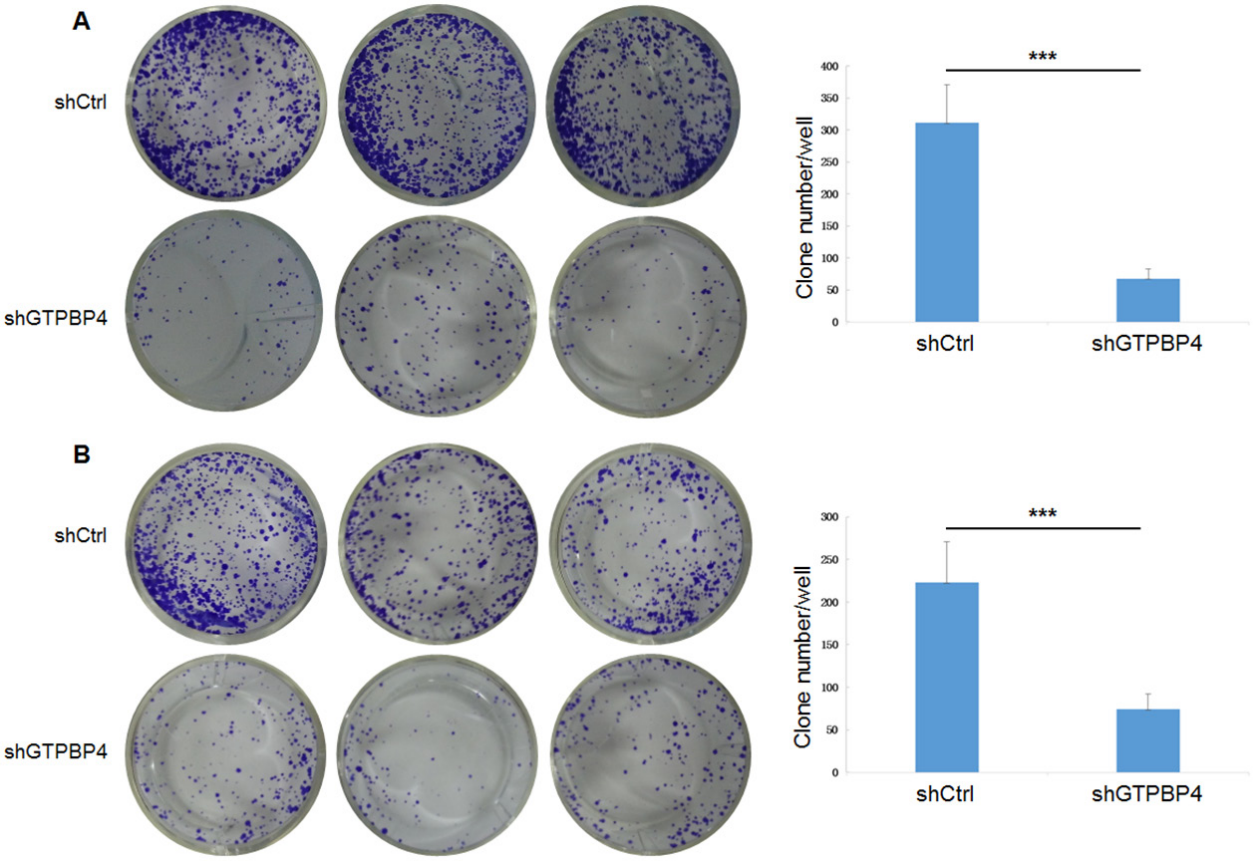
Knockdown of GTPBP4 reduced tumor colony formation in SMMC-7721 and HepG2 The tumor colony formation assay showed that knockdown of GTPBP4 reduced tumor colony formation both in **(A)** SMMC-7721 and **(B)** HepG2. Data were presented as means ± SD, ^***^P <0.001.

**Figure 6:**
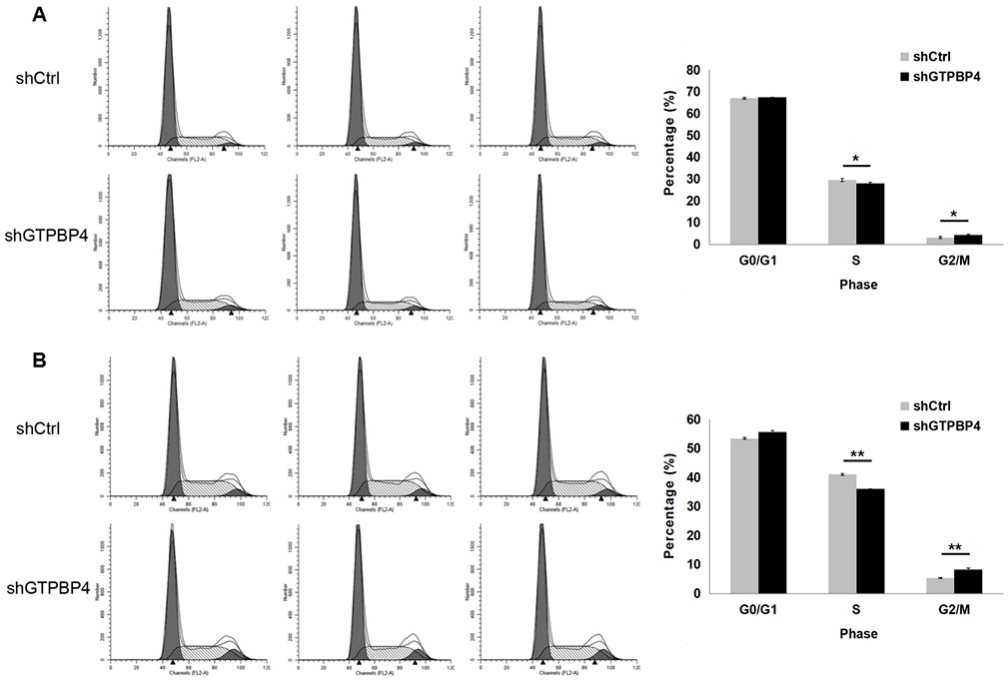
Knockdown of GTPBP4 induced cell cycle arrest in SMMC-7721 and HepG2 The percentages of cells in the G0/G1, S, G2/M phases were examined by FCM. G0/G1 and S phase populations decreased while the G2/M phase population increased in **(A)** SMMC-7721 and **(B)** HepG2 cells. Data were presented as means ± SD, ^*^P <0.05, ^**^P <0.01.

**Figure 7:**
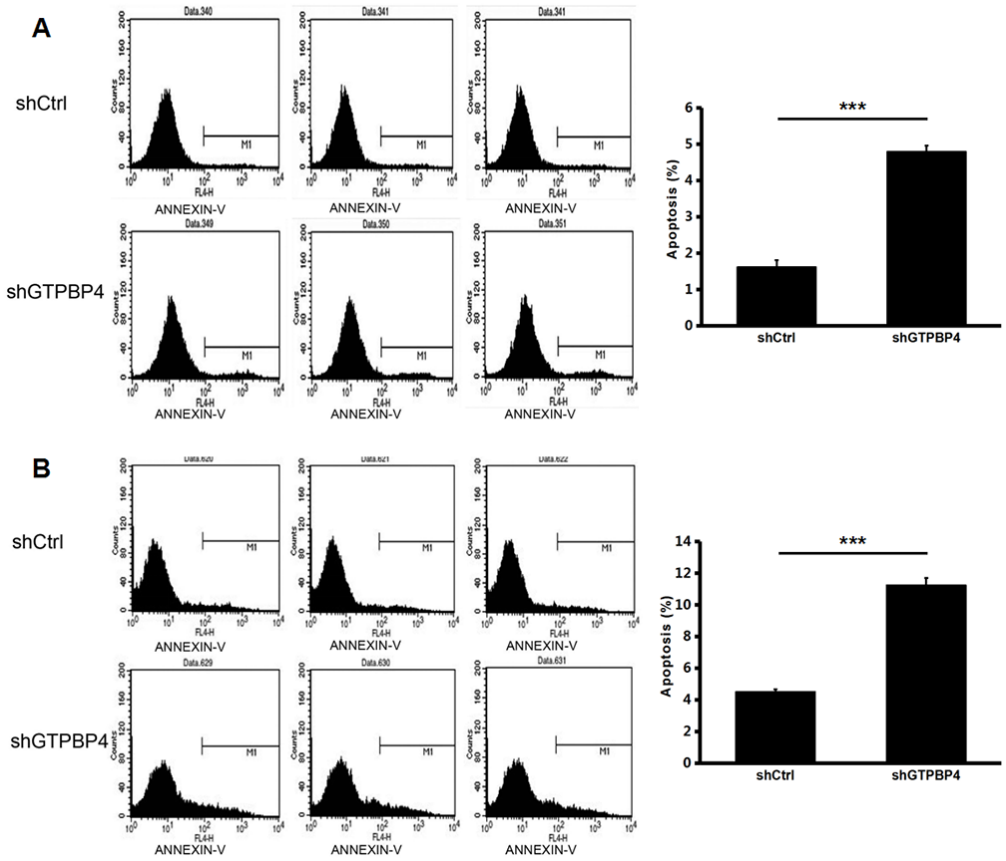
Knockdown of GTPBP4 increased cell apoptosis in SMMC-7721 and HepG2 Annexin V staining was measured by FCM to evaluate cell apoptosis. The percentage of apoptosis both in **(A)** SMMC-7721 and **(B)** HepG2 increased remarkably after GTPBP4 knockdown. Data were presented as means ± SD, ^***^P <0.001.

To examine the effects of GTPBP4 suppression on cancer cell proliferation, cellomics detection were conducted. As shown in Figure 4A, after shRNA lentivirus infections, proliferation clearly decreased in SMMC-7721 cells. And the cell count and its fold change also decreased in a time-dependent manner after shRNA lentivirus infections, reaching maximum inhibition on the 5th day (Figure 4B and 4C). We then conducted a tumor colony formation assay. As shown in Figure 5, the number of colonies was much lower after plating in shGTPBP4 groups compared to the shCtrl groups (*P* < 0.001) in two HCC cell lines. Flow cytometry (FCM) was then performed to examine the effects of suppression of GTPBP4 on cell cycle progression. The percentages of shGTPBP4 cells in the S phase of cell cycle were decreased, while the percentage in the G2/M phase was obviously increased, compared to shCtrl cells (Figure 6). This indicated that down-regulation of GTPBP4 increased cell cycle arrest at the G2/M phase. Moreover, the percentage of apoptotic cells dramatically increased after the shRNA lentivirus infection in both HCC cell lines (*P* < 0.001, Figure 7).

### Suppression of GTPBP4 attenuated tumor formation and growth of HCC *in vivo*

**Figure 8:**
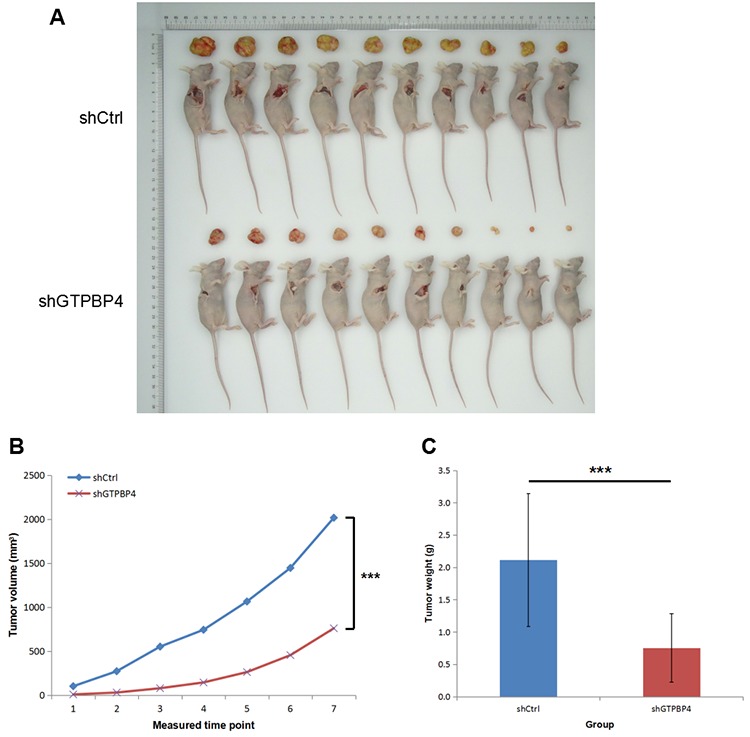
Knockdown of GTPBP4 inhibited tumorigenesis *in vivo* **(A)** Representative images of tumor size in BALB/c nude mice subcutaneously injected with control shRNA cells or GTPBP4 shRNA cells; **(B)** tumor volumes were calculated on each measured time point (every two days after xenograft establishment, for total 14 days and 7 measured time points); **(C)** tumor weight of xenografts were significantly reduced after GTPBP4 knockdown. Data were presented as means ± SD, ^***^P <0.001.

SMMC-7721 cells infected with lentiviruses expressing either control non-target shRNA or GTPBP4-shRNA were used and injected subcutaneously into nude mice (Supplementary Figure 2), and then the tumor volume and weight were examined. As shown in Figure 5A, the tumor size was significantly smaller in nude mice injected with cells infected with lentiviruses expressing GTPBP4-shRNA than that in the control group. The tumor volume was smaller in nude mice injected with GTPBP4-shRNA cells at all 7 time points (Figure 8B), and the tumor weight was also substantially lower in nude mice injected with GTPBP4-shRNA cells than that in the control group (Figure 8C).

### Differential genes and pathway enrichment analysis after GTPBP4 knockdown

**Figure 9:**
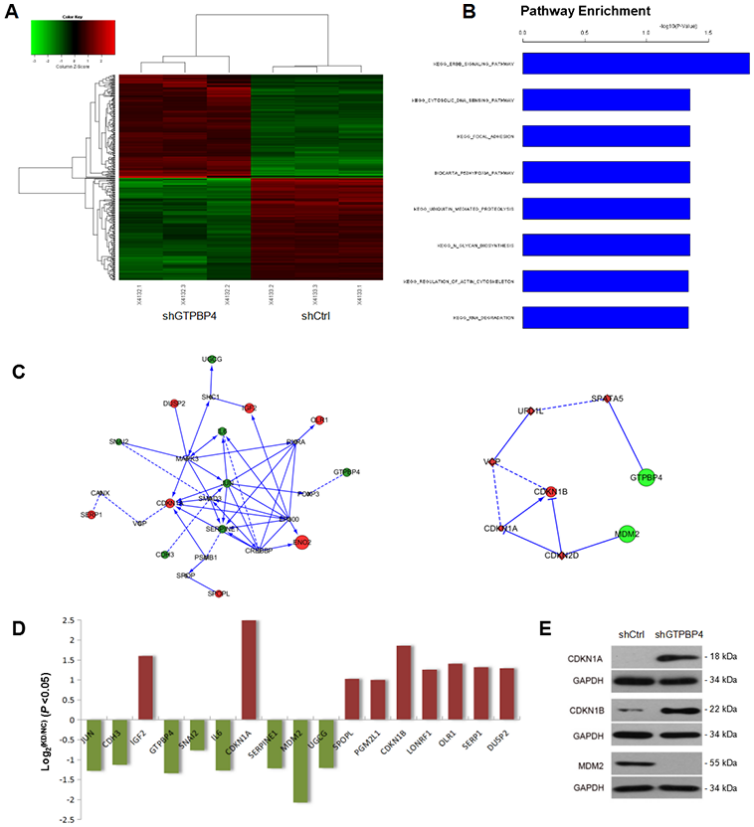
Comprehensive changes in gene expression and pathways pivotal to tumorigenesis in SMMC-7721 with GTPBP4 knockdown **(A)** Heatmap of 340 genes showed significant (*P* <0.05) differential expression (fold change >1) between cells transfected with GTPBP4 shRNA (green) and with control shRNA (red). Rows and columns represented genes and samples, respectively. A color scale for normalized expression data was shown in the upper left corner of the heatmap (green represented down-regulated genes and red represented up-regulated genes); **(B)** functional pathway enrichment of differentially expressed genes was analyzed based on IPA online software. Statistically significant modulations (*P* <0.001) of the top 8 pathways were shown. The statistical significance shown on the X axis was represented by the inverse log of the *P* value; **(C)** networks were constructed between GTPBP4 and select genes and pathway-related or down-stream genes, respectively. Green circles represented down-regulated genes, red circles represented up-regulated genes, and gray rhombuses represented linker genes. Solid arrows indicated confirmed regulatory relationships and dotted lines predicted regulatory relationships. Inhibitory relationships were indicated by “T” bars; **(D)** differentially expressed genes (*P* <0.05 and fold change >1) in network; **(E)** protein levels of select genes in SMMC-7721 cells transfected with negative control shRNA (shCtrl) or GTPBP4 shRNA (shGTPBP4) examined by western blot.

To further gain insights into the molecular mechanisms underlying the tumor suppression function of GTPBP4 knockdown in HCC, a microarray platform was used to compare the differential genes between the SMMC-7721 cells infected with lentivirus expressing either GTPBP4 shRNA or those with control shRNA. And a total of 340 genes showed significantly changed expression, with *P* < 0.05 and > 1.5 absolute value of fold change, including 168 upregulated genes and 172 downregulated genes (Figure 9A). Functional pathway enrichment of differentially expressed genes was analyzed based on IPA online software. Statistically significant modulations of the top 8 pathways were shown (*P* <0.001, Figure 9B). Among them, the ERBB signaling pathway is one of the largest differences in the pathway enrichment analysis. Moreover, networks were constructed between GTPBP4 and select genes and pathway-related or down-stream genes, respectively (Figure 9C). The mRNA levels of differentially expressed genes in networks were shown in Figure 9D. Additionally, the crucial genes CDKN1A, CDKN1B and MDM2, known as genes closely associated with cell growth and apoptosis, were checked by western blot (Figure 9E).

## DISCUSSION

Till now, the function and detailed underlying mechanism of GTPBP4 in malignant tumors have been poorly uncovered. A small number of existing studies have found a contradictory dual role of GTPBP4 in cancer. On one hand, Lee et al. [8] revealed that down-regulation and infrequent mutation of GTPBP4 were observed in human glioma cell lines and primary tumors. Upregulation of GTPBP4 after transfection could decrease the ability of Schwann cell growth, DNA synthesis and tumorigenicity. Further studies showed that GTPBP4 acted as a tumor suppressor gene by regulating Merlin and cyclin D1 to inhibit cell proliferation. On the other hand, some studies found that GTPBP4, as an oncogene, was upregulated in breast [12] and colorectal cancer [13] and high level of GTPBP4 was closely associated with unfavorable prognosis of these patients. Thus, in order to explore the exact role and function of GTPBP4 in HCC, the present study was done for the first time.

We first examined the expression levels of GTPBP4 mRNA and protein in HCC cell lines and tissues. Oncomine and MERAV databases were used to show that GTPBP4 mRNA was expressed higher in HCC tissues and cell lines than that in normal liver tissues. Afterward, four HCC cell lines and 90 cases of HCC tissues were chosen to verify the GTPBP4 mRNA and protein levels, respectively. The results also revealed that both mRNA and protein levels of GTPBP4 were significantly higher in HCC than those in normal liver cell line (L02) and HCC tissues. All these findings verified the high expression level of GTPBP4 in HCC.

Next, we explored the prognostic significance of GTPBP4 in HCC. The immunochemical results of 90 cases of HCC tissues were statistically analyzed. Chi-square test revealed that high expression of GTPBP4 was positively associated with higher TNM stage, which is widely believed to be responsible for the worse prognosis noted among patients with HCC. Moreover, Kaplan-Meier analysis of both our data and OncoLnc database showed that HCC patients with GTPBP4 high expression had poorer prognosis than those with GTPBP4 low expression. Additionally, both univariate and multivariate survival analyses strongly demonstrated that GTPBP4 was a significant independent adverse prognostic factor for patients with advanced HCC, which means GTPBP4 could be a potential prognostic biomarker or a molecular target for HCC.

Lastly, GTPBP4 functions and underlying molecular mechanisms in HCC were investigated systemically and in details with lentiviral-mediated shRNA strategy *in vitro* and *in vivo*. The results showed that GTPBP4 knockdown delayed cell proliferation, impaired colony formation ability, induced cell cycle arrest in G2/M period and promoted apoptosis in HCC cell lines. Besides, *in vivo* xenograft nude mice model revealed that GTPBP4 knockdown could significantly suppress HCC tumorigenesis. All these findings suggest that GTPBP4 serves as an oncogene and plays a pivotal role in HCC development. Moreover, gene microarray and further pathway enrichment analyses indicated that ERBB signaling pathway was the much more significantly changed one. Additionally, protein levels of the typically differential genes like CDKN1A, CDKN1B and MDM2 were identified by western blot. In fact, accumulating studies have found that ERBB signaling pathway is closely involved in the carcinogenesis [16-18], cell survival [19] and so on. CDKN1A is tightly controlled by the tumor suppressor protein p53, through which this protein mediates the p53-dependent cell cycle arrest [20-23]. CDKN1B encoded protein binds to and prevents the activation of cyclin E-CDK2 or cyclin D-CDK4 complexes, and thus controls the cell cycle progression [23-25]. And MDM2 mediates ubiquitination of p53/TP53, leading to its degradation by the proteasome inhibiting p53/TP53- and p73/TP73-mediated cell cycle arrest and apoptosis [26-29]. Recently, Lunardi et al. [30] have demonstrated that GTPBP4 knockdown induces p53 accumulation and activation in the absence of nucleolar disruption. In breast tumors with wild-type p53, increased expression of GTPBP4 correlates with reduced patient survival, emphasizing a potential relevance of this regulatory axis in cancer. Taken together, our results were consistent with those studies, which can at least partially explain the underlying mechanisms of GTPBP4 function in HCC.

However, some limitations of the study should be acknowledged. Firstly, in clinical analysis, it was a retrospective study with relatively small sample size, which is thus leading to a potential selection bias. Secondly, only one shRNA was designed and the corresponding results might be interfered by the off-target effects to some extent. Thirdly, although downstream differentially expressed genes and signaling pathways were screened out after GTPBP4 knockdown, the detailed regulatory mechanisms have not been fully elucidated. These issues will be further explored in our future experiments.

In conclusion, our findings revealed for the first time that GTPBP4 is overexpressed in HCC and regulates its cell survival and proliferation as an oncogene. More importantly, high GTPBP4 expression level significantly correlates with a poor prognosis in HCC patients. Therefore, GTPBP4 will be a potential therapeutic target or a biomarker for HCC.

## MATERIALS AND METHODS

### Patients and specimens

Human tissue microarray (TMA, catalog no. HLiv-HCC180Sur-04) was purchased from Shanghai Outdo Biotech, China, including 90 HCC tissues and the same amount of paired adjacent non-tumor tissues. The operations were carried out from August 2006 to November 2009, the last follow-up time was September 2013. Informed consent was obtained from all patients for the use of tissues in experimental procedures. This study was approved by the Ethics Committee of Anhui Provincial Hospital. All methods were performed in accordance with the guidelines and regulations of Anhui Provincial Hospital.

### Immunochemical staining and evaluation

Immunohistochemistry was performed with the GTPBP4 antibody at a dilution of 1:2000 according to a commercial protocol of Shanghai Outdo Biotech. Antibody staining was visualized with DAB and hematoxylin counterstain. The staining intensities of GTPBP4 were scored from 0 to 3 where 0 means negative, 1 weak, 2 moderate and 3 strong. The percentages of positively stained cells were scored in scales of 0 to 4, where 0 (0%), 1 (1-25%), 2 (26-50%), 3 (51-75%) and 4 (76-100%). The scores for percentages of positive cells and staining intensities were then multiplied to generate an immunoreactive score (IRS) for each case. The IRS ranged from 0-12. Cut-off levels for this scoring system were assigned as follows: high GTPBP4 expression was defined as an IRS of > 4; and low GTPBP4 expression was defined as an IRS of ≤ 4. These scores were assessed by two pathologists simultaneously.

### Cell culture

Human HCC cell lines (SMMC-7721, Huh7, Hep3B and HepG2) were obtained from the Cell Bank of the China Academy of Sciences (Shanghai, China). Cells were cultured in Dulbecco’s modified Eagle’s medium (DMEM, Gibco, Gaithersburg, USA) supplemented with 10% fetal bovine serum (FBS; HyClone Laboratories, Logan, USA), 100 U/ml penicillin and 100 μg/ml streptomycin (complete media). All cell cultures were maintained as a monolayer culture at 37 °C in a humidified atmosphere containing 5% CO_2_.

### Quantitative real-time PCR

Total RNA was extracted from SMMC-7721 or HepG2 cells using TRIzol^®^ RNA Isolation Reagent (Invitrogen, Carlsbad, CA) according to the manufacturer’s instructions. Reverse transcription was performed using the PrimeScript™ RT reagent kit (Takara, Dalian, China). All mRNA levels were normalized to the housekeeping gene GAPDH. The following GTPBP4 and GAPDH primers used in the study were listed in the Supplementary Table 1. All samples were treated under the same conditions and analyzed by qRT-PCR using SYBR Premix Ex Taq™ (Takara, Dalian, China) according to the manufacturer’s protocol. The cycling conditions were as follows: five minutes at 95 °C for preincubation, 45 amplification cycles of 15 seconds at 95 °C, 5 seconds at 95 °C, and 30 seconds at 60 °C. All experiments were carried out in triplicate.

### Western blot

Protein extracts from the HCC cell lines and Western blots were performed as described [31]. Equal amounts of proteins from HCC cell lines were subjected to western blot. Anti-GTPBP4 antibody and anti-GAPDH antibody (Abcam, UK) were used. All the information of main antibodies used in this study was listed in the Supplementary Table 2.

### Lentivirus construction and infection

To knock down GTPBP4 expression in cell lines, a recombinant lentiviral expression vector (pGSIL-shGTPBP4) containing a green fluorescent protein (GFP) tag was constructed. To generate lentiviral particles, the recombinant expression plasmid was co-transfected with a packaging plasmid system (psPAX2 and pMD2G) into SMMC-7721 cells, and viral particles were collected after 48 h. SMMC-7721 and HepG2 cells were infected with shGTPBP4 lentiviral vector or with a negative control (NC) vector without shGTPBP4 (shCtrl) for 24 h. The infection efficiency was preliminarily assessed in each experiment under a fluorescence microscope and then measured by sorting GFP-positive cells by flow cytometry (Beckman Coulter, USA). The stably infected cells were expanded and harvested for further experiments. The shGTPBP4 target sequence was as follows: GCTGGAGAGTATGACAGTGTActcgagTACACTGTCATACTCTCCAGC.

### Cell proliferation assay

SMMC-7721 cells infected with shGTPBP4 or shCtrl were plated in a 96-well plate at a density of 2000 cells in 100 μL per well and placed in a culture incubator at 37 °C and 5% CO_2_. After another 24 h of culture, cell numbers were autonomously quantified using the Cellomics ArrayScan VTI (Thermo, Rockford, IL, MA, USA) with a 488 nm laser once a day for a total of 5 days. Then, cell growth curves were produced for each condition.

### Colony formation assay

Cells infected with GTPBP4-shRNA lentivirus or NC lentivirus were seeded in 6-well plates at a density of 300-500 cells/well and further cultured in complete media for 10-15 days. After removal of the media and two rinses with PBS, the colonies were fixed with methanol for 15 min, stained with 0.1% crystal violet for 10 min and photographed using a digital camera (Leica, Germany). Experiments were repeated three times.

### Cell cycle and apoptosis

SMMC-7721 and HepG2 cells infected with shGTPBP4 lentiviral vector or with a negative control (NC) vector without shGTPBP4 (shCtrl) were harvested at 48 hr. After double staining with FITC-Annexin V and Propidium iodide (PI) using the Annexin V-FITC Apoptosis Detection Kit (BD Biosciences) according to the manufacturer’s recommendations, the cells were analyzed by flow cytometry (FACScan^®^; BD Biosciences) equipped with the CellQuest software (BD Biosciences) as previously described [31]. Cells were discriminated into viable, dead, early apoptotic or apoptotic cells, and the relative amounts of early apoptotic cells were compared to shCtrl. Cells for cell cycle analysis were stained with PI using the CycleTEST™ PLUS DNA Reagent Kit (BD Biosciences) following the manufacturer’s protocol and analyzed by FACScan. The percentage of cells in G0/G1, S and G2/M phase were counted and compared.

### HCC subcutaneous tumor model in nude mice

Six-week-old female BALB/c nude mice were purchased from the animal center of the Cancer Institute of the Chinese Academy of Medical Science. All experimental procedures were carried out according to the National Institutes of Health guide for the care and use of Laboratory animals and complied with ARRIVE guidelines. The SMMC-7721 subcutaneous model was established as previously described [31]. The mice were randomly divided into 2 groups (10 mice per group), a GTPBP4 knockdown group and a mock group. The tumor size was measured with a caliper every other day, and tumor volume was calculated using the formula: volume = length × width^2^/2. At the end of a 14-day observation period, the mice were sacrificed, and tumor tissues were collected for formalin fixation and preparation of paraffin-embedded sections for immunohistochemistry.

### Differential genes and pathway enrichment analysis after GTPBP4 knockdown

Gene chips were used for path array analysis and meta-analysis with IPA online software. Signal Histogram, Relative Signal Box Plot, and Pearson’s correction signal confirmed the quality of these analyses. Classical pathways and related genes were analyzed using IPA (www.ingenuity.com).

### Statistical analysis

All statistical analyses were performed using SPSS 19.0 for Windows (SPSS, Inc., Chicago, IL, USA). Quantitative data were presented as mean ± standard deviation (SD). Pearson chi-square test or Fisher’s exact test was used to analyze the correlation between GTPBP4 expression and clinicopathological parameters. The Kaplan-Meier method and the log-rank test were used for survival analysis. Cox regression model was used for multivariate survival analysis to identify prognostic factors that were significant in the univariate analysis. A *P* value < 0.05 was considered as statistically significant.

## SUPPLEMENTARY MATERIALS FIGURES AND TABLES



## References

[1] Siegel RL, Miller KD, Jemal A (2017). Cancer Statistics, 2017. CA Cancer J Clin.

[2] Llovet JM, Zucman-Rossi J, Pikarsky E, Sangro B, Schwartz M, Sherman M, Gores G (2016). Hepatocellular carcinoma. Nat Rev Dis Primers.

[3] Yang JD, Roberts LR (2010). Hepatocellular carcinoma: a global view. Nat Rev Gastroenterol Hepatol.

[4] Schulze K, Imbeaud S, Letouzé E, Alexandrov LB, Calderaro J, Rebouissou S, Couchy G, Meiller C, Shinde J, Soysouvanh F, Calatayud AL, Pinyol R, Pelletier L (2015). Exome sequencing of hepatocellular carcinomas identifies new mutational signatures and potential therapeutic targets. Nat Genet.

[5] Fujimoto A, Furuta M, Totoki Y, Tsunoda T, Kato M, Shiraishi Y, Tanaka H, Taniguchi H, Kawakami Y, Ueno M, Gotoh K, Ariizumi S, Wardell CP (2016). Whole-genome mutational landscape and characterization of noncoding and structural mutations in liver cancer. Nat Genet.

[6] Laping NJ, Olson BA, Zhu Y (2001). Identification of a novel nuclear guanosine triphosphate-binding protein differentially expressed in renal disease. J Am Soc Nephrol.

[7] Grupe A, Li Y, Rowland C, Nowotny P, Hinrichs AL, Smemo S, Kauwe JS, Maxwell TJ, Cherny S, Doil L, Tacey K, van Luchene R, Myers A (2006). A scan of chromosome 10 identifies a novel locus showing strong association with late-onset Alzheimer disease. Am J Hum Genet.

[8] Lee H, Kim D, Dan HC, Wu EL, Gritsko TM, Cao C, Nicosia SV, Golemis EA, Liu W, Coppola D, Brem SS, Testa JR, Cheng JQ (2007). Identification and characterization of putative tumor suppressor NGB, a GTP-binding protein that interacts with the neurofibromatosis 2 protein. Mol Cell Biol.

[9] Park JH, Jensen BC, Kifer CT, Parsons M (2001). A novel nucleolar G-protein conserved in eukaryotes. J Cell Sci.

[10] Jensen BC, Wang Q, Kifer CT, Parsons M (2003). The NOG1 GTP-binding protein is required for biogenesis of the 60 S ribosomal subunit. J Biol Chem.

[11] Honma Y, Kitamura A, Shioda R, Maruyama H, Ozaki K, Oda Y, Mini T, Jenö P, Maki Y, Yonezawa K, Hurt E, Ueno M, Uritani M (2006). TOR regulates late steps of ribosome maturation in the nucleoplasm via Nog1 in response to nutrients. EMBO J.

[12] van de Vijver MJ, He YD, van’t Veer LJ, Dai H, Hart AA, Voskuil DW, Schreiber GJ, Peterse JL, Roberts C, Marton MJ, Parrish M, Atsma D, Witteveen A (2002). A gene-expression signature as a predictor of survival in breast cancer. N Engl J Med.

[13] Yu H, Jin S, Zhang N, Xu Q (2016). Up-regulation of GTPBP4 in colorectal carcinoma is responsible for tumor metastasis. Biochem Biophys Res Commun.

[14] Shaul YD, Yuan B, Thiru P, Nutter-Upham A, McCallum S, Lanzkron C, Bell GW, Sabatini DM (2016). MERAV: a tool for comparing gene expression across human tissues and cell types. Nucleic Acids Res.

[15] Anaya J (2016). OncoLnc: linking TCGA survival data to mRNAs, miRNAs, and lncRNAs. PeerJ Computer Science.

[16] Bai Y, Xue Y, Xie X, Yu T, Zhu Y, Ge Q, Lu Z (2014). The RNA expression signature of the HepG2 cell line as determined by the integrated analysis of miRNA and mRNA expression profiles. Gene.

[17] Berasain C, Avila MA (2014). The EGFR signalling system in the liver: from hepatoprotection to hepatocarcinogenesis. J Gastroenterol.

[18] Scheving LA, Zhang X, Stevenson MC, Weintraub MA, Abbasi A, Clarke AM, Threadgill DW, Russell WE (2015). Loss of hepatocyte ERBB3 but not EGFR impairs hepatocarcinogenesis. Am J Physiol Gastrointest Liver Physiol.

[19] Chen JY, Chen YJ, Yen CJ, Chen WS, Huang WC (2016). HBx sensitizes hepatocellular carcinoma cells to lapatinib by up-regulating ErbB3. Oncotarget.

[20] Aravinthan A, Mells G, Allison M, Leathart J, Kotronen A, Yki-Jarvinen H, Daly AK, Day CP, Anstee QM, Alexander G (2014). Gene polymorphisms of cellular senescence marker p21 and disease progression in non-alcohol-related fatty liver disease. Cell Cycle.

[21] Marhenke S, Buitrago-Molina LE, Endig J, Orlik J, Schweitzer N, Klett S, Longerich T, Geffers R, Sánchez Muñoz A, Dorrell C, Katz SF, Lechel A, Weng H (2014). p21 promotes sustained liver regeneration and hepatocarcinogenesis in chronic cholestatic liver injury. Gut.

[22] Yano M, Ohkoshi S, Aoki YH, Takahashi H, Kurita S, Yamazaki K, Suzuki K, Yamagiwa S, Sanpei A, Fujimaki S, Wakai T, Kudo SE, Matsuda Y, Aoyagi Y (2013). Hepatitis B virus X induces cell proliferation in the hepatocarcinogenesis via up-regulation of cytoplasmic p21 expression. Liver Int.

[23] Kim JH, Choi YK, Byun JK, Kim MK, Kang YN, Kim SH, Lee S, Jang BK, Park KG (2016). Estrogen-related receptor γ is upregulated in liver cancer and its inhibition suppresses liver cancer cell proliferation via induction of p21 and p27. Exp Mol Med.

[24] Wang J, Huo K, Ma L, Tang L, Li D, Huang X, Yuan Y, Li C, Wang W, Guan W, Chen H, Jin C, Wei J (2011). Toward an understanding of the protein interaction network of the human liver. Mol Syst Biol.

[25] Mitselou A, Karapiperides D, Nesseris I, Vougiouklakis T, Agnantis NJ (2010). Altered expression of cell cycle and apoptotic proteins in human liver pathologies. Anticancer Res.

[26] Wu B, Chu X, Feng C, Hou J, Fan H, Liu N, Li C, Kong X, Ye X, Meng S (2015). Heat shock protein gp96 decreases p53 stability by regulating Mdm2 E3 ligase activity in liver cancer. Cancer Lett.

[27] Tang T, Song X, Yang Z, Huang L, Wang W, Tan H (2014). Association between murine double minute 2 T309G polymorphism and risk of liver cancer. Tumour Biol.

[28] Wang X, Zhang X, Qiu B, Tang Y, Sun H, Ji H, Liu Y, Shi L, Song G, Yang Y (2012). MDM2 SNP309T>G polymorphism increases susceptibility to hepatitis B virus-related hepatocellular carcinoma in a northeast Han Chinese population. Liver Int.

[29] Embade N, Fernández-Ramos D, Varela-Rey M, Beraza N, Sini M, Gutiérrez de Juan V, Woodhoo A, Martínez-López N, Rodríguez-Iruretagoyena B, Bustamante FJ, de la Hoz AB, Carracedo A, Xirodimas DP (2012). Murine double minute 2 regulates Hu antigen R stability in human liver and colon cancer through NEDDylation. Hepatology.

[30] Lunardi A, Di Minin G, Provero P, Dal Ferro M, Carotti M, Del Sal G, Collavin L (2010). A genome-scale protein interaction profile of Drosophila p53 uncovers additional nodes of the human p53 network. Proc Natl Acad Sci U S A.

[31] Wang W, Jia WD, Hu B, Pan YY (2017). RAB10 overexpression promotes tumor growth and indicates poor prognosis of hepatocellular carcinoma. Oncotarget.

