# Supplementary material for: Knockdown of GTPBP4 inhibits cell growth and survival in human hepatocellular carcinoma and its prognostic significance
Source: Oncotarget. 2017 Oct 5;8(55):93984–97. doi: 10.18632/oncotarget.21500 (PMC5706849; doi:10.18632/oncotarget.21500)
Supplement: Supplementary file 1 [file oncotarget-08-93984-s001.pdf]

Knockdown of GTPBP4 inhibits cell growth and survival in human hepatocellular carcinoma and its prognostic significance

SUPPLEMENTARY MATERIALS

Supplementary Table 1: The primer sequences for RT-PCR

Gene	Forward primer (5'-3')	Reverse primer (5'-3')	Product (bp)
GTPBP4	CAGCCCTATGCGTTCACAAC	TCCCAGGAGTGTCTACAACCT	88
GAPDH	TGACTTCAACAGCGACACCCA	CACCCTGTTGCTGTAGCCAAA	121

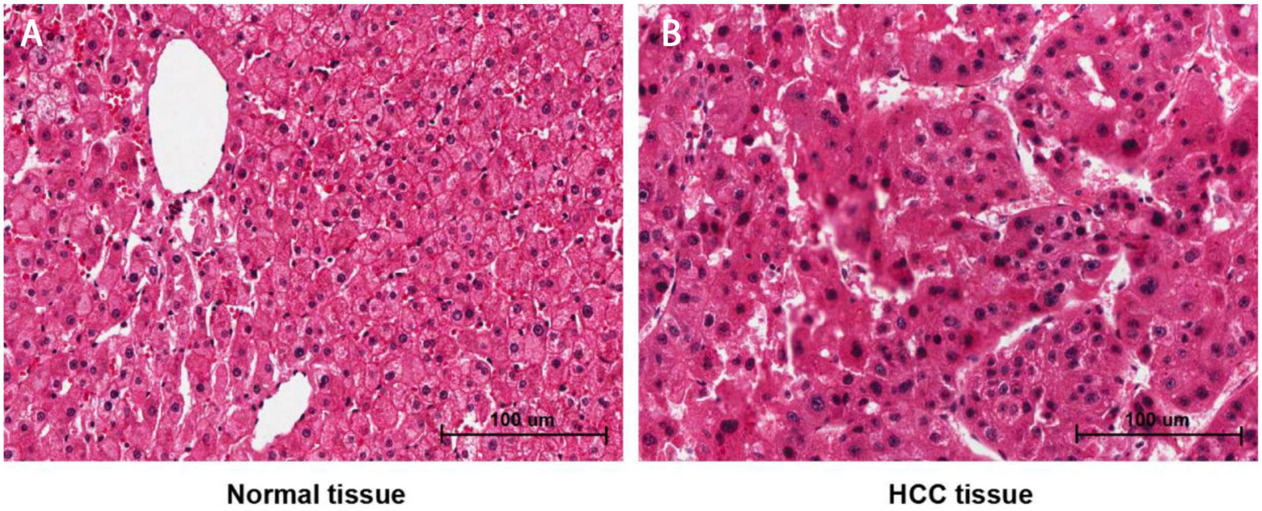

Supplementary Figure 1: Examples of HE staining of HCC and adjacent normal tissues. **(A)**: Normal tissue; **(B)**: HCC tissue; magnified 200X, corresponding to Figure 1E.

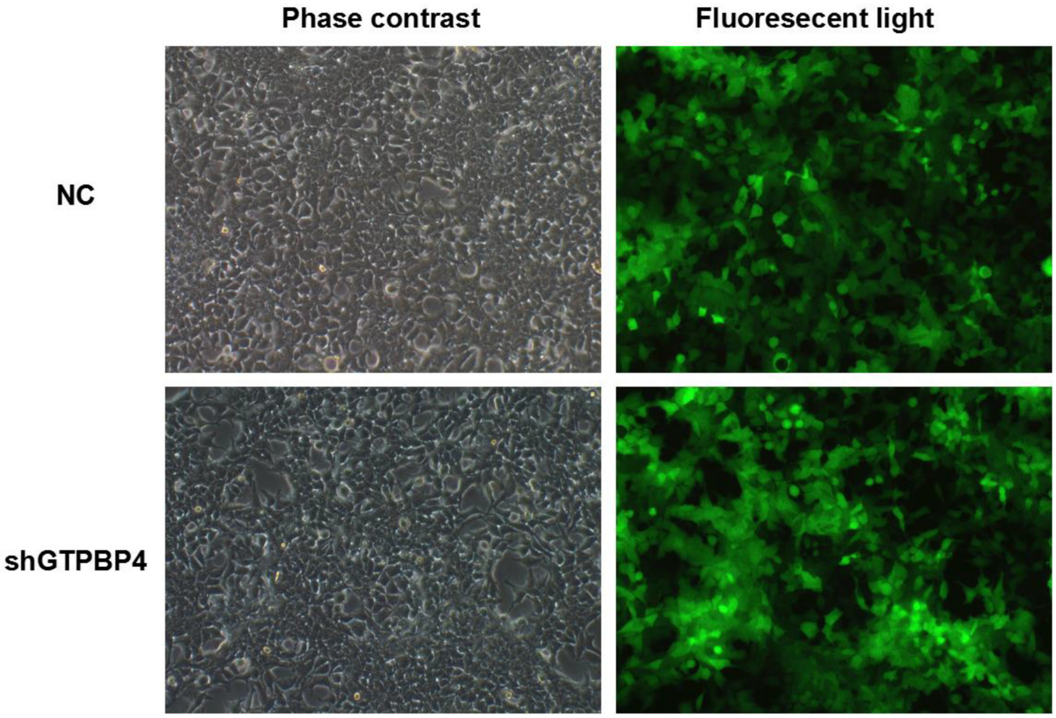

Supplementary Figure 2: The efficiency of lentivirus infection. SMMC-7721 cells were selected by puromycin for 48h after infection, and examined by fluorescence and light microscopy. More than 85% of the cells were expressed GFP (magnified 100X).

Supplementary Table 2: Primary and secondary antibodies used in the study

Name	Species	Company	Cat No.	Dilution
GTPBP4	Rabbit	Abcam	ab184124	1/2000
CDKN1B	Rabbit	CST	3686	1/500
CDKN1A	Rabbit	Abcam	ab7960	1/500
MDM2	Rabbit	Abcam	ab38618	1/200
GFP	Mouse	Santa Cruz	sc-9996	1/4000
GAPDH	Mouse	Santa Cruz	sc-32233	1/5000
rabbit IgG		Santa Cruz	sc-2004	1:5000
mouse IgG		Santa Cruz	sc-2005	1:5000
Goat IgG		Santa Cruz	sc-2005	1:2000
